# Investigation of Reducing Interface State Density in 4H-SiC by Increasing Oxidation Rate

**DOI:** 10.3390/nano13091568

**Published:** 2023-05-06

**Authors:** Shuai Li, Jun Luo, Tianchun Ye

**Affiliations:** 1 Institute of Microelectronics of the Chinese Academy of Sciences, Beijing 100029, China; lishuai@ime.ac.cn (S.L.); tcye@ime.ac.cn (T.Y.); 2 University of Chinese Academy of Sciences, Beijing 100049, China

**Keywords:** 4H-SiC, oxidation rate, interface states density, phosphorus implantation, SIMS, HRTEM, STEM, EDS

## Abstract

Detailed investigations of the pre-oxidation phosphorus implantation process are required to increase the oxidation rate in 4H-SiC metal-oxide-semiconductor (MOS) capacitors. This study focuses on the SiO_2_/SiC interface characteristics of pre-oxidation using phosphorus implantation methods. The inversion channel mobility of a metal-oxide-semiconductor field effect transistor (MOSFET) was decreased via a high interface state density and the coulomb-scattering mechanisms of the carriers. High-resolution transmission electron microscopy (HRTEM) and scanning transmission electron microscopy (STEM) were used to evaluate the SiO_2_/SiC interface’s morphology. According to the energy-dispersive X-ray spectrometry (EDS) results, it was found that phosphorus implantation reduced the accumulation of carbon at the SiO_2_/SiC interface. Moreover, phosphorus distributed on the SiO_2_/SiC interface exhibited a Gaussian profile, and the nitrogen concentration at the SiO_2_/SiC interface may be correlated with the content of phosphorus. This research presents a new approach for increasing the oxidation rate of SiC and reducing the interface state density.

## 1. Introduction

In the field of power devices, the most commercialized material used to replace silicon is 4H-SiC, primarily because 4H-SiC possesses unique physical properties, such as a high critical breakdown electric field and high electron mobility. However, the carrier mobility of SiC metal-oxide-semiconductor field effect transistors (MOSFETs) without annealing technology was only approximately 5 cm^2^/Vs [1] due to the high interface trap density. Theoretically, for Si and SiC MOSFET devices with the same breakdown voltage, the on-resistance of the SiC device is two orders of magnitude lower than that of the Si device at a given breakdown voltage [2]. Therefore, the SiO_2_/SiC interface state density (Dit) must be reduced, and the performance of MOSFETs must be improved. The main advantage of SiC over other wide-bandgap semiconductors is its native oxide SiO_2_, which can be grown via thermal oxidation. The thermal oxidation process may be regarded as the origin of interface traps and near-interface oxide traps, which limit the channel mobility and reliability of MOSFETs [3,4]. Furthermore, Coulomb-scattering and trapping/de-trapping mechanisms also reduce device mobility [5,6]. Chung et al. first reported high-temperature nitric oxide (NO) annealing for SiC MOSFETs [7]. Post-oxidation during NO annealing is the most established process for forming high-quality oxides on 4H-SiC with lower interface traps. Nitrogen can passivate the SiO_2_/SiC interface and decompose carbon clusters [8]. Other nitrogen-containing gasses’ annealing conditions (N_2_O and NH_3_) have also been widely used in academic research and device fabrication. However, excess nitrogen can cause device threshold voltage instability; thus, it is necessary to reasonably regulate the nitrogen content at the SiO_2_/SiC interface [9,10]. Annealing under a high temperature and low partial pressure of oxygen can reduce the SiO_2_/SiC interface carbon content and decrease interface defects, but it also causes a negative flatband voltage shift [11]. High-temperature H_2_ pretreatment was performed on a SiC surface before oxidation film growth, which was followed by a chemical vapor deposition SiO_2_ film process [12]. This method can significantly reduce interface state density and improve device mobility. However, this method required high process equipment capabilities, and H_2_ is prone to explosion at a high temperature. Kobayashi proposed a new oxidation method whereby the SiC surface is with H_2_ and then passivated in a N_2_ atmosphere. Reportedly, the interface state density was reduced to 10^10^ cm^−2^ eV^−1^ near the SiC conduction band energy level [13], but the oxidation film defects decreased the breakdown electric field. Rapid thermal annealing in inert gas (Ar or N_2_) after thermal oxidation was also a commonly used process for decreasing interface defects [14]; the annealing temperature was generally the same as the oxidation temperature, and this process step was considered to be able to remove the excess carbon at the SiO_2_/SiC interface and in the oxidation film, but there was no direct evidence of carbon diffusion. Moderate argon annealing could improve the oxidation film’s reliability, but the reduction in the interface state density and improvement in device mobility were still limited. Nitrogen or oxygen plasma, or a mixture of both, can reduce the SiO_2_/SiC interface’s sub-oxide content, but this process method also caused significant flatband voltage instability [15,16]. Kosugi et al. reported that when annealing in a wet atmosphere, a device’s mobility increased to 40 cm^2^/Vs [17]. Using ethyl silicate (TEOS) as a precursor to deposit SiO_2_ as a dielectric film, or when depositing a SiO_2_ film through the low-temperature chemical vapor deposition process, the interface state density of the low-temperature chemical vapor deposition process was poorer than that of the thermal oxidation process, and the low-temperature process caused a negative flatband voltage [18,19,20]. When silicon oxide and high-dielectric constant films are grown using the atomic layer deposition (ALD) process, as has been widely studied [21,22,23], they can enhance the breakdown electric field. However, the low-temperature ALD process produced more dielectric film/SiC interface defects, and post-annealing was required to reduce these interface defects. Thermal SiO_2_ and ALD high-dielectric-constant film stacked structures were also used to reduce the interface state density; however, this processing method produced many defects within the oxidation film, resulting in a significant flatband voltage hysteresis phenomenon [24,25,26]. Inspired by N passivation, Okamoto et al. passivated the interface states with POCl3 gas, and the peak mobility increased to 101 cm^2^/Vs [27]. However, in the phosphorus passivation process, phosphorus was uniformly distributed in the SiO_2_, and several charges were introduced into silicon oxide, causing a large flatband voltage. A similar conclusion was reported by Yano et al. [28]. The doping process plays an important role in semiconductor device manufacturing, as monolayer doping has attracted much attention due to its great potential with respect to the conformal doping of non-planar nanostructures [29]. Uniform doping to realize ultra-shallow junctions has been key in the efforts to achieve device scaling, and this spin on the dopant process can satisfy these requirements [30,31,32]. In addition to the mentioned doping techniques, ion implantation technology and the evaporation process were also widely used in the fabrication of Si devices and SiC devices. Lichtenwalner et al. injected small amounts of alkaline earth elements into silicon carbide before oxidation, which increased the mobile carrier concentration and improved mobility [33]. Phosphorus or oxygen were also injected into SiC to reduce the number of interface states, but a larger dosage caused lattice damage [34,35]. In this study, we propose a pre-oxidation phosphorus implantation process that can decrease the interface state density by controlling the pretreatment conditions without causing additional lattice damage.

## 2. Materials and Methods

**Figure 1:**
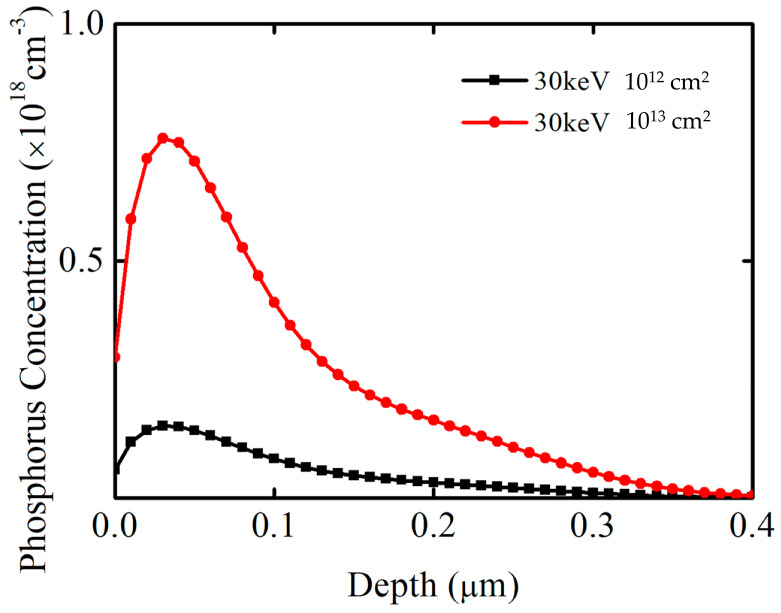
Silvaco Technology Computer−Aided Design simulation results under different pretreatment conditions. The horizontal axis represents the phosphorus implantation depth, and the vertical axis represents the phosphorus concentration.

The samples used in this study included commercially available n-type epitaxial 4H-SiC wafers that were (4.0 ± 0.5)° off-axis toward <11–20>. The MOS capacitors were fabricated on the (0001) plane face. The thickness of the epilayer was 10 µm, and the concentration was (5.06–10.62) × 10^15^ cm^−3^. First, the standard Radio Corporation of America (RCA) cleaning process was adopted for wafer cleaning. This process was employed to remove contamination, which was followed by the growth of a sacrificial oxide film. The native oxide layer and sacrificial oxide layer were removed using a diluted 10% HF solution. HF is highly corrosive and reacts with silicon oxide to form fluorine silicide. The ammonia solution used in the standard Radio Corporation of America cleaning process reacts with fluorine silicide, and the sacrificial oxide layer can be completely removed. The purpose of forming the sacrificial oxide layer was to remove damage and defects on the SiC’s surface and reduce SiO_2_/SiC interface defects. Second, a field oxide film was grown via chemical vapor deposition, which was followed by phosphorus implantation. A sample without implantation was also included for comparison. It is crucial to reasonably select the phosphorus implantation conditions. A larger amount of energy leads to a wider implantation profile, meaning the elements cannot be completely consumed by oxidation. A small amount of energy cannot promote SiC oxidation. A Silvaco Technology Computer-Aided Design (TCAD) simulation was used to determine the pretreatment conditions, for which the results are shown in Figure 1. The implantation energy was 30 keV, and the implantation dosages were 10^12^ cm^2^ and 10^13^ cm^2^. The target oxide film thickness was 50 nm, and the peak value of the implantation depth was approximately 25 nm. The relationship between the thickness of consumed SiC and the thickness of SiO_2_ was 1:2.17; therefore, simulated pre-oxidation implantation conditions met the requirements.

Subsequently, the wafers were introduced into an O_2_ atmosphere tube at 1400 °C for 13 min, wherein some samples underwent annealing at 1200 °C for 70 min. All samples were grown with polysilicon in an in situ doping low-pressure chemical vapor deposition process, which is a process of forming gate electrodes. Both metal electrodes, i.e., backside and frontside, were Al electrodes that were all grown through a sputtering process. The thicknesses of the Al electrodes were all 1 µm. Table 1 shows the experimental conditions determined based on the implantation simulation. The sample without pretreatment was used as the reference group, and the sample annealed after oxidation was also added for comparison. Keysight B1505A (San Francisco, CA, USA) equipment was used to measure the samples’ electrical characteristics. High-resolution transmission electron microscopy was used to characterize the SiO_2_/SiC interface’s morphology. Using a Thermo Scientific (Waltham, CA, USA) Helios G4 HX Dual Beam focused ion beam/scanning electron microscopy machine, the sample was prepared using in situ focused ion beam lift-out technology. The electron beam deposition of platinum (Pt) and ion beam deposition of platinum (Pt) were used to protect the sample surface from ion beam damage.

Transmission electron microscopy images were captured using a Thermo Scientific (Waltham, CA, USA) Themis Z spherical-aberration-corrected transmission electron microscope with a 200KV accelerating voltage. Bright-field scanning transmission electron microscopy images and high-angle annular dark-field scanning transmission electron microscopy images were used to further characterize the SiO_2_/SiC interface morphology. Bright-field scanning transmission electron microscopy images and high-angle annular dark-field scanning transmission electron microscopy images were photographed using a Thermo Scientific Themis Z spherical-aberration-corrected transmission electron microscope operated at an accelerating voltage of 200 KV. Energy-dispersive X-ray spectrometry was used to analyze the concentration distribution of carbon elements and oxygen elements at the SiO_2_/SiC interface. Energy-dispersive X-ray spectrometry line analysis data were obtained using the Super X FEI (Hillsboro, OR, USA) system in scanning transmission electron microscopy mode. Secondary ion mass spectroscopy was also used to characterize the concentration distribution of nitrogen and phosphorus at the SiO_2_/SiC interface, and the analytical data were obtained using cesium cluster mode.

## 3. Results and Discussion

**Figure 2:**
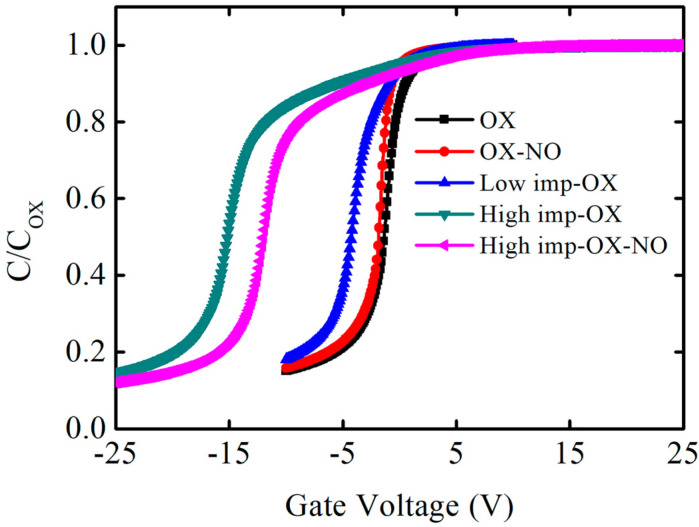
Capacitance−voltage characteristics of the fabricated capacitors structures at 1 MHz. The horizontal axis represents the gate voltage, and the vertical axis represents the normalized result of the measured capacitance on the oxidation film.

The capacitance–voltage characteristics of the MOS capacitors are shown in Figure 2. When the high-frequency capacitance–voltage curve was measured, a direct current bias voltage was applied at both electrodes of the capacitor structure, and an alternating current signal was used to measure the capacitance value. The alternating current signal frequency was 1 MHZ, and the direct current voltage step was 0.1 V. The direct current voltage caused the capacitor characteristics to shift from the positive voltage accumulation region to the negative voltage depletion region, and high-frequency capacitance–voltage curves were obtained at 1 MHZ. In Figure 2, the horizontal axis represents the direct current voltage, while the vertical axis represents the normalized results. C_OX_ represents the oxidation film’s capacitance, which was obtained via the accumulation region capacitance value. The oxidation film thickness can be obtained via Formula (1). The flatband capacitance can be obtained from Formula (2), where the flatband capacitance corresponds to flatband voltage. A is the area of the metal gate electrode, which is 2 × 10^4^ cm^2^; $\varepsilon_{0}$ is the vacuum dielectric constant, which is 8.85 × 10^−14^ F/cm; $\varepsilon_{s}$ is the relative dielectric constant of the oxidation film, which is 3.9; and $t_{ox}$ is the oxidation film thickness. *L* is the debye length, which is 3.93 × 10^−6^ cm in our experiment; $\varepsilon_{sic}$ is the relative dielectric constant of SiC, which is 9.7.

(1) $$C_{ox}=A\times \frac{\varepsilon_{0}\varepsilon_{s}}{t_{ox}}$$

(2) $$C_{FB}=\frac{1}{1+\frac{L\varepsilon_{s}}{t_{ox}\varepsilon_{sic}}}$$

**Figure 3:**
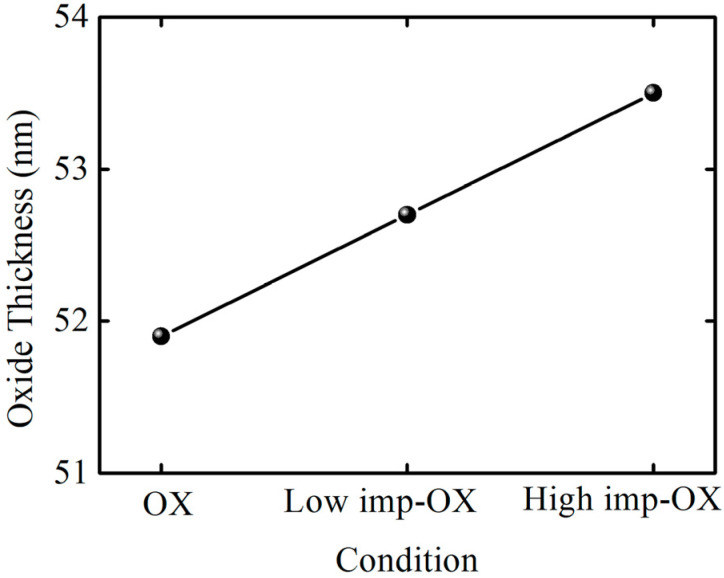
Three conditions’ oxidation film thicknesses.

One phenomenon can be distinctly observed in the capacitance–voltage curves: pre-implantation before oxidation can increase the oxidation film’s thickness within the same oxidation time, indicating that the SiC oxidation rate increases with the implantation dosage. Figure 3 shows the oxidation film thickness variation trend under different pretreatment conditions; in order to evaluate the effect of pretreatment on oxidation rate, OX, low-imp-OX, and high-imp-OX samples were selected for analysis. Thus, it was determined that the oxidation film thickness was the lowest under the OX condition, whereas the oxidation film thickness increased after the pretreatment. Thus, the SiC oxidation rate can be correlated with the pretreatment conditions. The increase in oxidation film thickness is associated with an increase in the oxidation rate of ion-damaged or amorphized SiC [36,37]. The pretreatment increased the SiC oxidation rate, which may be related to the fact that the results were interpreted using a modified Deal–Grove model. Increasing the amount of phosphorus led to an increase in the linear rate constant factor and the parabolic rate constant factor. The increase in the linear rate constant was attributed to defects from the doping-induced lattice mismatch, and the increase in the diffusion-limited parabolic rate constant was attributed to the degradation of the oxidation film’s quality originating from the doping-induced lattice mismatch; pre-implantation oxidation increased the activation energy of the SiC oxidation reaction, resulting in higher oxidation reaction activity [38]. The increased activation energy was attributed to the higher phosphorus concentration, as the higher phosphorus concentration induced the amorphized SiC crystal phenomenon, and it can be assumed that the chemical bonding was more covalent in the amorphous phase than in the crystalline phase [39]. In Figure 3, it can be seen that the high-imp-ox sample had a faster oxidation rate, and we believe that the activation energy of the oxidation reaction in this sample group was higher during the SiC oxidation reaction, wherein chemical bonds were more covalent in the amorphous phase than in the crystalline phase.

In Figure 2, another phenomenon can be observed: the capacitance–voltage curves of the pretreatment samples shifted significantly to the left, and the flatband voltage was more negative, indicating that the shift values were also related to the implantation dosage, while the high phosphorus concentration introduced more positive charges and the shift value was more pronounced. These findings are similar to the results concerning nitrogen-related gas passivation. Compared to the OX condition sample, the flatband voltage of the OX-NO condition sample shifted slightly to the left, indicating that nitrogen passivation can cause hole traps. As discussed by S. DIMITRIJEV et al., excess nitrogen at the SiO_2_/SiC interface resulted in a larger flatband voltage shift [40], and excess nitrogen introduced more positive charges. It can also be observed from the capacitance–voltage curves in Figure 2 that the flatband voltage of the OX-NO sample has shifted to the left compared to that of the OX sample. Note that the flatband voltage is determined by the metal-semiconductor work function difference $\emptyset_{MS}$, the fixed charge $Q_{f}$, the interface traps $Q_{it}$, the mobile ions $\rho_{m}$, and the oxide traps $\rho_{ot}$, as shown in Formula (3). *C_OX_* represents the oxidation film capacitance, and $t_{ox}$ is the position of trap charges in the oxidation film. The left shift of the flatband voltage is attributed to phosphorus fixed charges or phosphorus mobile ions. The introduced positive charges caused a relatively negative flatband voltage. The large number of positive charges caused by the pretreatment dominated the effect on the flatband voltage, and the negative flatband voltage shift was more significant. Some samples were annealed with nitric oxide gas after oxidation to further reduce the SiO_2_/SiC interface defects; however, compared to the high-imp-OX sample, the leftward flatband voltage shift of the high-imp-OX-NO sample was not very significant, which can be explained as follows: when the nitrogen passivated the SiO_2_/SiC interface, there were still traps in the oxidation film, and thus electrons were captured by oxidation traps, causing the flatband voltage to shift to the right [41]. Annealing in a nitric oxide atmosphere can reduce the interface state density and flatband voltage shift.

(3) $$V_{FB}=\emptyset_{MS}-\frac{Q_{f}}{C_{ox}}-\frac{Q_{it}}{C_{ox}}-\frac{1}{C_{ox}}\int_{0}^{t_{ox}}\rho_{m}\left(x\right)dx-\frac{1}{C_{ox}}\int_{0}^{t_{ox}}\rho_{ot}\left(x\right)dx$$

**Figure 4:**
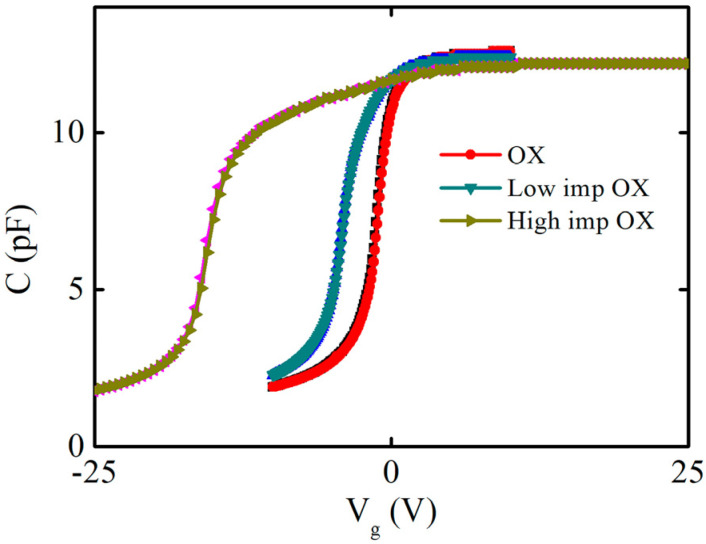
Capacitance−voltage hysteresis characteristics of different samples. The pink and brown curves are the results of the capacitance-voltage hysteresis characteristic of the high−imp−OX sample. The blue and green curves are the results of the capacitance-voltage hysteresis characteristic of the low−imp−OX sample. The red and black curves are the results of the capacitance-voltage hysteresis characteristic of the OX sample.

To confirm the phosphorus-positive charge types, the capacitance–voltage hysteresis characteristics were identified, and the capacitance–voltage hysteresis curves were scanned; firstly, the capacitance–voltage curve was scanned from the negative voltage depletion region to the positive voltage accumulation region, and then the capacitance–voltage curve was scanned from the positive voltage accumulation region to the negative voltage depletion region. The voltage-scanning frequency was 1 MHZ, and the voltage step was 0.1 V. Figure 4 shows the capacitance–voltage hysteresis characteristics of the samples under different implantation conditions (OX, low-imp-OX, and high-imp-OX). Mobile charges drift during the forward- and reverse-scanning process, causing a shift in the flatband voltage. The forward and reverse capacitance–voltage curves of the three condition samples were approximately coincident, with no significant difference found. Phosphorus charges do not exhibit mobile behavior and are more likely to be fixed charges.

The distribution of interface state density (Dit) and energy levels was obtained via the high-frequency (1 MHz) and quasi-static method, with a voltage sweep step of 0.1 V. The quasi-static capacitance–voltage characteristic measurement involves the application of a very slow (<0.1 V/S) time-varying voltage signal that linearly increases over time to the gate electrode while simultaneously using a sensitive ammeter to measure the current flowing through the capacitor. Based on the amplitude of the changes in the gate current and gate voltage, the quasi-static capacitance can be obtained, which can be calculated using Formula (4), where $I_{g}$ is the gate current, $V_{g}$ is the gate voltage, and *t* is the measurement time. When the capacitor structure is in a quasi-static state, the measurement results of the low-frequency signal are the same as the quasi-static state results.

(4) $$C=\frac{I_{g}}{\frac{dV_{g}}{dt}}$$

**Figure 5:**
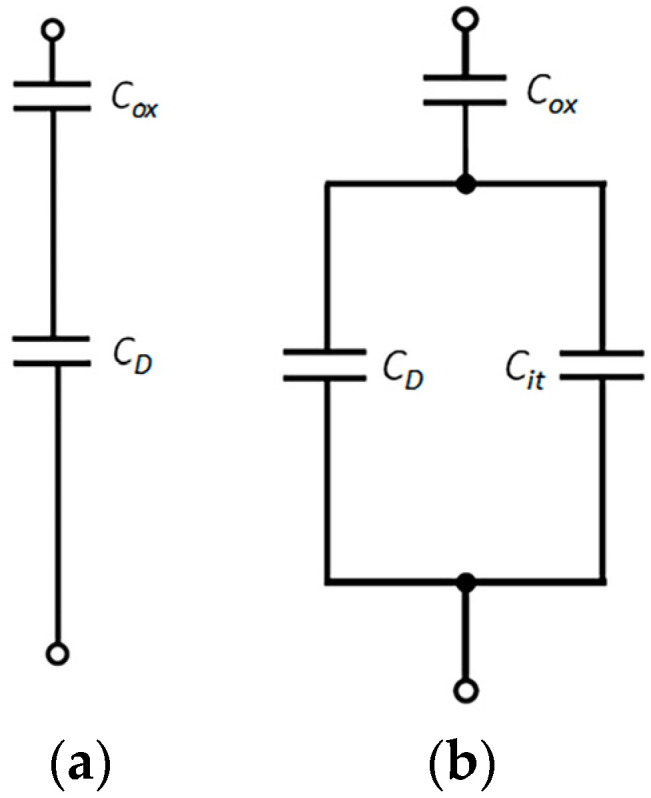
(**a**) High-frequency equivalent circuit and (**b**) quasi-static equivalent circuit.

The equivalent circuit of the capacitor structure under a high-frequency signal and quasi-static signal is shown in Figure 5. Cox is the oxidation film capacitance and *C_D_* is the semiconductor space-charge-region capacitance. When the metal electrode of the capacitor is loaded with a high-frequency signal, the charging and discharging of the interface states cannot keep pace with the voltage signal, and the capacitance related to the interface states does not contribute to the high-frequency capacitance. When the applied direct current bias voltage and the applied signal frequency change slowly, the charging and discharging of the interface states always keep pace with the change in the voltage signal. Thus, the influence of the interface states’ capacitance must be considered, as shown in Figure 5b, where Cit is the interface states’ capacitance of the low-frequency equivalent circuit. In Figure 5b, the equivalent capacitance at a low frequency was obtained as Formula (5), and the equivalent capacitance at a high frequency was obtained as Formula (6).

(5) $$\frac{1}{C_{LF}}=\frac{1}{C_{OX}}+\frac{1}{C_{D}+C_{it}}$$

(6) $$\frac{1}{C_{HF}}=\frac{1}{C_{ox}}+\frac{1}{C_{D}}$$

**Figure 6:**
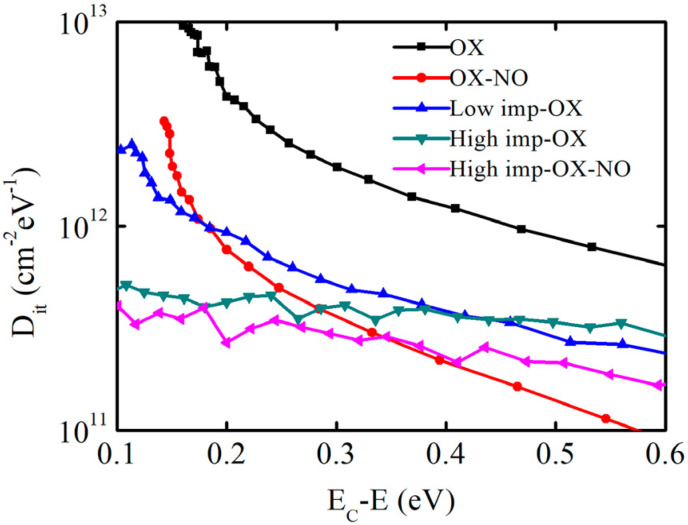
Energy level distribution of interface state density extracted via a high−frequency (1 MHz) quasi-static method.

The results of the interface state density measurements at room temperature for the 4H-SiC MOS capacitors with and without implantation are shown in Figure 6. EC is the SiC conduction band energy level. It is worth mentioning that the Dit values from the high-frequency/quasi-static capacitance–voltage measurements may be uncertain at EC; as a result, annealing in an N atmosphere after implantation oxidation induces fast interface states. However, these do not influence the effect of phosphorus implantation oxidation. Therefore, the obtained values are considered reliable and exhibit a certain trend. The interface state density of the samples under OX, low-imp-OX, and high-imp-OX conditions gradually decreased near the SiC conduction band energy level (Ec-E ≤ 0.3 eV), and significant advantages can be observed compared to the sample without the pretreatment (OX-NO). The high interface state density near the SiC conduction band energy is the main factor affecting device mobility [42], but at the deep energy level position (Ec-E > 0.4 eV), the interface state density under the high-imp-ox condition was higher than that under the low-imp-ox condition. For the high-imp-ox-NO condition, the interface state density was significantly higher than that under the OX-NO condition when Ec-E > 0.3 eV; this may be due to the higher pretreatment dosage changing the distribution of nitrogen content at the SiO_2_/SiC interface, which reduced the nitrogen passivation effects at the SiO_2_/SiC interface.

The advantage of phosphorus pretreatment oxidation is that it can reduce the SiO_2_/SiC interface state density, but phosphorus fixed charges also cause a significant flatband voltage shift; thus, this process method cannot be used in actual device fabrication.

**Figure 7:**
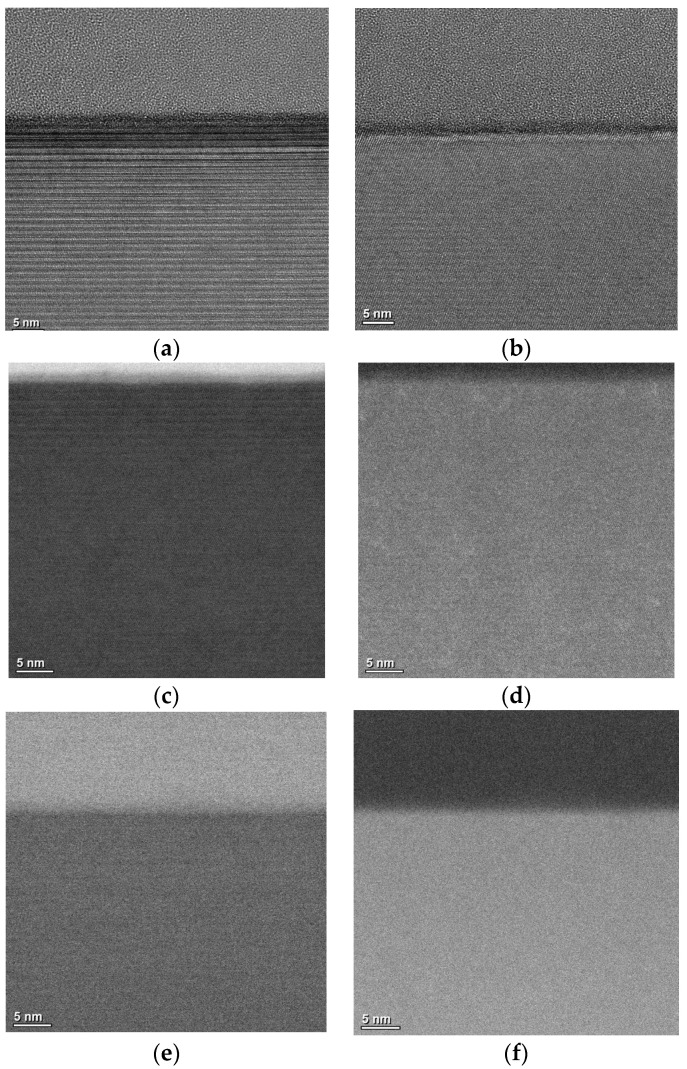
(**a**) High-resolution transmission electron microscopy image of the SiO_2_/4H-SiC interface of sample without pretreatment; (**b**) high-resolution transmission electron microscopy image of the SiO_2_/4H-SiC interface of the pretreated sample; (**c**) bright-field scanning transmission electron microscopy image of the SiO_2_/4H-SiC interface of sample without pretreatment; (**d**) high-angle annular dark-field scanning transmission electron microscopy image of the SiO_2_/4H-SiC interface of sample without pretreatment; (**e**) bright-field scanning transmission electron microscopy image of the SiO_2_/4H-SiC interface of pretreated sample; (**f**) high-angle annular dark-field scanning transmission electron microscopy image of the SiO_2_/4H-SiC interface of pretreated sample.

**Figure 8:**
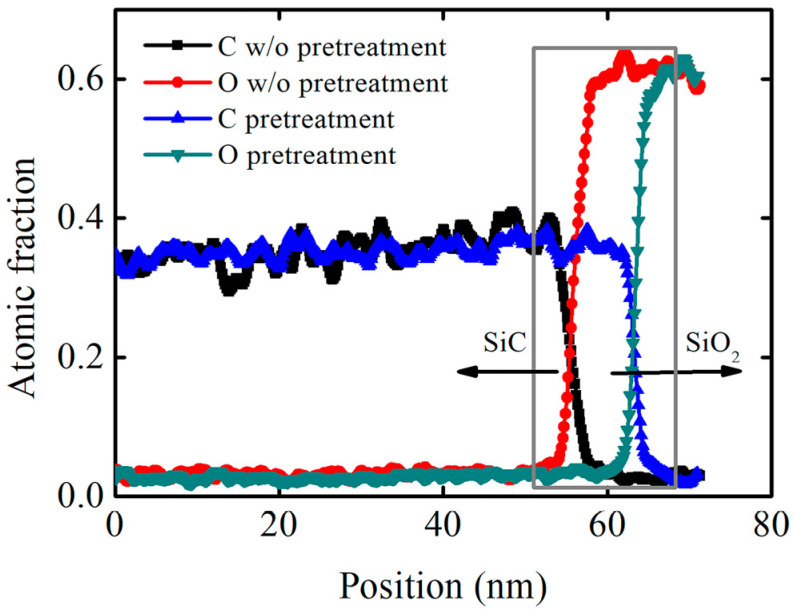
The distribution of carbon and oxygen fractions characterized via energy−dispersive X−ray spectrometry. Arrows represent the SiC and SiO_2_ respectively.

Pretreatment causes a certain degree of damage to the SiC crystal structure, and it can increase SiC’s oxidation rate. In order to further analyze the mechanism of decreasing interface state density, high-resolution transmission electron microscopy and scanning transmission electron microscopy were used to observe the SiO_2_/SiC interface morphology. A sample without pretreatment was also added for comparison, and the OX-NO sample and high-imp-OX-NO sample were used to evaluate the SiO_2_/SiC interface morphology, for which the characterization results are shown in Figure 7. SiO_2_ was amorphous, which was consistent with the findings of other research reports [43]. For the sample that was not pretreated, a significant abnormal contrast was found on the SiC side, as shown in Figure 7a. However, for the pretreated sample, no abnormal contrast was found (as demonstrated in Figure 7b), and this conclusion is also valid for multidirectional tilting. Therefore, we confirmed the complexity of SiC oxidation, in which suboxides could have been formed at the SiO_2_/SiC interface [44,45]; this conclusion has also been reported in other studies [46]. Figure 7c–f shows bright-field scanning transmission electron microscopy (BFSTEM) images and high angle annular dark field scanning transmission electron microscopy (HAADF STEM) images. From Figure 7c,d, it can be seen that there may be elements that overlap with the region at the SiO_2_/SiC interface. Energy-dispersive X-ray spectrometry (EDS) confirmed the carbon and oxygen distribution at the SiO_2_/SiC interface, for which the results are shown in Figure 8. For the pretreatment sample, the carbon and oxygen content distribution profiles of SiO_2_/SiC were steeper. The carbon concentration rapidly decreased from the SiC side to the SiO_2_ side. However, for the sample without pretreatment, the carbon distribution at the SiO_2_/SiC interface was spread over a wider distance, and the carbon concentration slowly decreased from the SiC side to SiO_2_ side. For the pretreated sample, the overlapping distance of the carbon and oxygen fractions at the SiO_2_/SiC interface was approximately 3 nm, whereas the overlapping distance of the carbon and oxygen components at the SiO_2_/SiC interface for the sample without pretreatment was approximately 6 nm. From the above discussion, it can be concluded that phosphorus pretreatment oxidation can improve the oxidation rate and alleviate carbon accumulation at the SiO_2_/SiC interface, which can explain the decrease in the interface state density of the pretreatment sample shown in Figure 6. For the pretreated sample, no excess lattice damage was found on the SiC side, indicating that the pretreatment process was reasonable.

**Figure 9:**
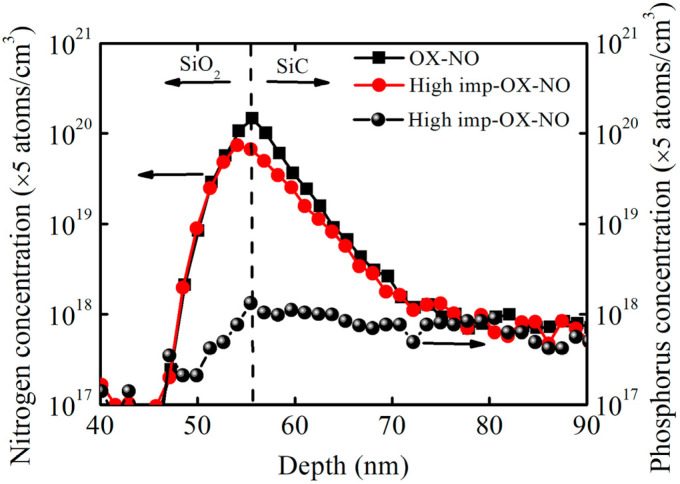
Depth profile of nitrogen atoms and phosphorus atoms for SiO_2_/SiC structures obtained via secondary ion mass spectroscopy. The arrow pointing to the left indicates the nitrogen concentration and SiO_2_, and the arrow pointing to the right indicates the phosphorus concentration and SiC.

To evaluate the distribution of phosphorus atoms near the SiO_2_/SiC interface, secondary-ion mass spectroscopy measurements were taken after growing SiO_2_ and annealing it in NO at high temperatures. Figure 9 shows the depth profiles for the concentrations of nitrogen atoms and phosphorus atoms in the samples with and without implantation (the high-imp-OX-NO sample and OX-NO sample). Owing to the use of the same NO annealing conditions, the OX-NO and high-imp-OX-NO samples were expected to exhibit the same nitrogen concentration profiles. Evidently, inside the oxide material, the depth profiles for nitrogen atoms were nearly identical in the OX-NO and high-imp-OX-NO samples. However, at the interface, the density of the nitrogen atoms was approximately two times higher in the sample that underwent only NO annealing than in the sample with phosphorus implantation. This finding suggests that the depth profile of the nitrogen concentration may be dependent on the phosphorus concentration. Inside the oxide material, the concentration of the phosphorus atoms increased along with the oxide depth, reaching a maximum at the interface and exhibiting an approximate Gaussian distribution at the SiO_2_/SiC interface. For the high-imp-OX-NO samples, the lower nitrogen atom concentration at the SiO_2_/SiC interface may be the reason for the higher interface state density at the deep-energy level, as shown in Figure 6.

## 4. Conclusions

This study demonstrated that the nitrogen–phosphorus hybrid passivation technique can effectively reduce the interface state density at the SiO_2_/SiC interface. The pretreatment increased the oxidation rate but caused a significant flatband voltage shift. Moreover, high-resolution transmission electron microscopy analysis and scanning transmission electron microscopy were used to evaluate the SiO_2_/SiC interface’s morphology. For the sample without pretreatment, abnormal contrast was found at the SiO_2_/SiC interface, thus confirming the complexity of SiC oxidation; however, for the pretreated sample, no abnormal contrast was found at the SiO_2_/SiC interface. Through energy-dispersive X-ray spectrometry, it was determined that implantation reduced the overlapping distance between the carbon and oxygen fractions at the SiO_2_/SiC interface and alleviated the accumulation of carbon at the SiO_2_/SiC interface. Moreover, pretreatment does not cause additional SiC lattice damage. For the high-imp-OX-NO sample, secondary ion mass spectroscopy analysis revealed that the implanted phosphorus atoms may change the concentration of nitrogen atoms at the SiO_2_/SiC interface and that the lower nitrogen atom concentration at the SiO_2_/SiC interface may be the reason for the higher interface state density at the deep energy level. A limitation of phosphorus oxidation was that it caused a large flatband voltage drift, and future work can focus on improving the flatband voltage stability.

## Figures and Tables

**Table 1:** Plan of the pre-implantation oxidation experiment.

	Implantation Condition		Oxide Condition		NO Annealing Condition	
	Dosage (cm^2^)	Energy (keV)	Temperature (°C)	Time (min)	Temperature (°C)	Time (min)
OX	-	-	1400	13	-	-
OX-NO	-	-	1400	13	1200	70
Low-imp-OX	10^12^	30	1400	13	-	-
High-imp-OX	10^13^	30	1400	13	-	-
High-imp-OX-NO	10^13^	30	1400	13	1200	70

## Data Availability

Not applicable.
